# An Internet of Things and Fuzzy Markup Language Based Approach to Prevent the Risk of Falling Object Accidents in the Execution Phase of Construction Projects

**DOI:** 10.3390/s21196461

**Published:** 2021-09-27

**Authors:** María Martínez-Rojas, María José Gacto, Autilia Vitiello, Giovanni Acampora, Jose Manuel Soto-Hidalgo

**Affiliations:** 1 Department of Economics and Business Management, School of Industrial Engineering, University of Málaga, 29016 Málaga, Spain; mmrojas@uma.es; 2 Department of Computer Science, University of Jaen, 23071 Jaén, Spain; mgacto@ujaen.es; 3 Department of Physics “Ettore Pancini”, University of Naples Federico II, 80126 Naples, Italy; autilia.vitiello@unina.it (A.V.); giovanni.acampora@unina.it (G.A.); 4 Department of Computer Architecture and Technology, University of Granada, 18011 Granada, Spain

**Keywords:** construction projects, execution phase, safety, falling objects, Internet of Things, Fuzzy Markup Language

## Abstract

The Internet of Things (IoT) paradigm is establishing itself as a technology to improve data acquisition and information management in the construction field. It is consolidating as an emerging technology in all phases of the life cycle of projects and specifically in the execution phase of a construction project. One of the fundamental tasks in this phase is related to Health and Safety Management since the accident rate in this sector is very high compared to other phases or even sectors. For example, one of the most critical risks is falling objects due to the peculiarities of the construction process. Therefore, the integration of both technology and safety expert knowledge in this task is a key issue including ubiquitous computing, real-time decision capacity and expert knowledge management from risks with imprecise data. Starting from this vision, the goal of this paper is to introduce an IoT infrastructure integrated with JFML, an open-source library for Fuzzy Logic Systems according to the IEEE Std 1855-2016, to support imprecise experts’ decision making in facing the risk of falling objects. The system advises the worker of the risk level of accidents in real-time employing a smart wristband. The proposed IoT infrastructure has been tested in three different scenarios involving habitual working situations and characterized by different levels of falling objects risk. As assessed by an expert panel, the proposed system shows suitable results.

## 1. Introduction

Information and Communication Technology (ICT) has been slow to adapt to the construction industry for several reasons, although they are mainly related to the sector’s own characteristics [1], e.g., the temporality of the projects, the traditional character, the simultaneousness of works, the singularity of each construction project. However, a major transformation concerning the use of new technologies to support the management of construction projects has been observed in the last decade [2].

The life cycle of construction projects includes several phases: (i) requirements identification, (ii) project planning, (iii) design (iv) execution, (v) maintenance and (vi) demolition. Among these phases, the two that take the longest period of time in the life cycle of a building project are the execution and the maintenance phases [3]. From a management perspective, the objective in each one of these phases is different and it requires different tools. For example, in the early stages of the project, coordination and collaboration between the different involved parties is essential, while in the execution phase the main focus is on cost, schedule, quality and safety control [4]. In the maintenance phase, it is essential to control various factors such as air quality, energy performance and building maintenance, among others. However, all these phases share the need for an adequate data acquisition process as well as techniques to manage such data properly [5]. In recent decades, a revolutionary technology named Building Information Modeling (BIM) has provided an opportunity to move to the new digital era enabling automation capabilities for more integrated communication, data exchange and sharing between project actors. In this sense, architects, engineers, and construction experts can efficiently plan, design, build and manage buildings. Such a concept of BIM is commonly known as passive BIM. However, when the efficiency of building alternatives is required to be evaluated during the design or the construction phase, the passive BIM approach may not be sufficient. Dynamic systems, which connect BIM with analytical functions or solutions with optimisation techniques to improve the decision making process, are necessary [6].

In this context, data capture can be facilitated by the diversity of existing sensors, as well as by communication among them, whereas efficient information extraction and processing are made possible by the application of different artificial intelligence techniques that can provide intelligent support in the life cycle of construction projects.

This vision has been enhanced by the paradigm known as the Internet of Things (IoT), which describes a system of interrelated and uniquely identified computing devices that have the ability to transfer data over a network, without requiring human interactions [7]. Recently, this paradigm is being considered by researchers in the field of construction, especially in the execution and maintenance phase through the sensorization of physical spaces [8] and wearable sensing devices (WSDs). Internet of Things brings, among other aspects, remarkable benefits in the construction life cycle, distinguishing among others the reduction of costs, smarter designs and knowledge about the environment in real-time, etc. [9]. However, one of the aspects that has recently attracted most interest in the scientific community and that represents a great challenge is related to safety [10,11,12,13.

On the other hand, the use of computational intelligence techniques and intelligent systems, which are able to solve complex and multidisciplinary problems in an automatic way from the data provided by the Internet of Things paradigm, supporting the decisions of an expert, are also contributing to great benefits in the field of construction. In particular to provide intelligent support on the prevention of accidents at any of the construction phases. Nevertheless, the data provided by the IoT framework are usually affected by uncertainty and, in order to support any decision-making process, the human behavior or expert knowledge have to be modeled. To achieve this goal, Fuzzy Logic Systems (FLSs), which are rule-based expert systems based on fuzzy set theory to represent the semantics of rules and to process inference when input data are provided [14], have been successfully used in a wide range of real-world problems in the construction sector. For example, for risk construction management [15,16,17], for rehabilitation and maintenance tasks [18,19,20], tracking materials and decision-making in construction management [21,22,23], or sustainability [24]. In addition, fuzzy systems have also been applied to modelling of uncertainties related to occupational risks in the construction sector [25,26,27]. FLS can include a priori expert knowledge and represent systems for which it is not possible to obtain a mathematical model.

In this context, the IEEE Computational Intelligence Society (IEEE-CIS) has sponsored the publication of the new standard for FLS (IEEE Std 1855-2016) [28]. This standard was established with the main objective of providing the fuzzy community with a unique and well-defined tool allowing a system to be designed completely independently from the specific hardware/software. The new standard defines a new W3C eXtensible Markup Language (XML) based language, named Fuzzy Markup Language (FML) [29] aimed at providing a unified and well-defined representation of interoperable FLS [30]. Additionally, in order to make the IEEE standard operative and useful for the fuzzy community, the library JFML [31] has been developed to offer a complete implementation of the new IEEE standard. Some hardware/sensor developments based on JFML have also been successfully done [32].

Therefore, the integration of data capture technologies in some phases of the life cycle of a construction project with both the Internet of Things paradigm and the use of Fuzzy Logic Systems poses an interesting challenge that can provide significant benefits for the prevention of accidents in the phases of a construction project in real-time. These aspects have motivated the present work by the idea of defining an IoT infrastructure as the core of ubiquitous computing and automatic data transfer from sensors with the use of JFML to monitoring the risk of falling objects accidents in some phases of the life cycle of a construction project. Specifically, we propose to use the MQTT protocol to send data from sensors over the defined IoT architecture and to integrate it with the JFML library to represent FLS in a human language to model expert knowledge in the prevention of falling objects accidents in the execution phase. To test our architecture, an expert FLS modeled according to the IEEE std 1855-2016 is used. The input variables of this FLS are associated with the most commonly used sensors in the execution phase to prevent accidents, whereas the output variable models the risk level of falling objects accident with four degrees: low, medium, high and very high. As shown in the experimental session, the results obtained by our proposal are assessed suitable by an expert panel.

The paper is structured as follows. In Section 2, some preliminary concepts related to the most existing common tasks and risks in the execution phase of the life cycle of a construction project, the Internet of Things and the Fuzzy Markup Language are presented in a general way. In Section 3, the potential technologies used in the execution phase are presented, from the point of view of the Internet of Things, while in Section 4 an IOT-JFML-based system for the management of data collected by the sensors in the task of prevention the risk of falling object accidents is developed. An illustrative case study focused on a building project and the execution phase where expert knowledge in the falling object accidents prevention is detailed in Section 5. Finally, the main conclusions and future works are reflected in Section 6.

## 2. Preliminaries

The new paradigms based on emerging technologies can provide significant advantages in the construction sector for different important tasks in the life cycle of construction projects. Specifically, we will focus on the execution phase, which is the one that can derive the most benefits from these technologies. Therefore, in this section, firstly, the main important task to be considered in the execution phase of the life cycle of construction projects are described (Section 2.1). Secondly, the main risks existing in the execution phase are analyzed (Section 2.2). Finally, in Section 2.3 and Section 2.4, we detail the main aspects of the Internet of Things paradigm and the fuzzy markup language and JFML for applying computational intelligence techniques to the data provided by the Internet of Things paradigm, respectively.

### 2.1. Execution Phase of the Life Cycle in the Construction Field

The life cycle of construction projects includes several phases: (i) requirements identification, (ii) project planning, (iii) design (iv) execution, (v) maintenance and (vi) demolition. Within these phases, the execution phase takes the longest period of time in the life cycle of a building [3]. From a management perspective, in the execution phase, the main tasks are generally focused on cost, schedule, quality and safety control. The following tasks are of interest during the project implementation:

- Monitoring of resources and materials. The monitoring and tracking of equipment and materials can bring important benefits for the execution of the process, achieving a reduction in the deadlines as well as in the costs of the work [33].
- Communication and collaboration. Management during the construction process could be improved using real-time communication and collaboration with devices that integrate building information modeling (BIM) and IoT technologies [34]. Currently, a new approach named “active building information modeling” is emerging in the construction community, connecting optimization techniques and BIM [6,35].
- Performance of the construction process and progress monitoring. This aspect can leverage these technologies in several ways. First, to know the current status of the construction process in real-time. On the other hand, to synchronize the construction planning based on the monitoring of the [36] process.
- Health and Safety Management. This task is fundamental in construction sites since the accident rate in this sector is very high compared to other sectors. In this sense, the use of technologies that allow monitoring or detecting possible risks in real-time can be of great interest to avoid or reduce the number of accidents [37].

### 2.2. Risks in the Execution Phase

Over the years, many studies have been conducted to understand the types and causes of accidents inherent to the construction industry. The most common accidents during the execution phase are: crane accidents, slips, trips and falls, electrical/heat/chemical burns, falls from heights, scaffold accidents, but the type of accident with the highest risk is falling objects [38].

“Being hit by dashing and flitting objects”, “being hit by objects”, “falling objects” and “being stung by something” on a worksite can expose workers to relatively minor injuries, such as cuts and abrasions, as well as more serious injuries, such as concussions or blindness. Working beneath scaffolds or other areas where overhead work is being performed puts workers at risk from falling objects [39]. Flying objects become a concern when workers are using power tools or performing tasks that involve pushing, pulling or prying [40].

Among the main causes of accidents are: insufficient PPE (head protection for workers and visitors), unprotected roof edges, absence of covered walkways or fan scaffolds, and excessive winds [40,41,42,43]. The types of injuries commonly sustained in construction accidents involving falling objects are: lacerations, bruises, broken bones, neck and back injuries, concussions and traumatic brain injuries, spine injuries and paralysis, permanent disabilities and death.

For the reasons stated in this section, our proposal focuses on the risk of accidents due to falling objects during the construction process.

### 2.3. Internet of Things

The concept of the Internet of Things (IoT) refers to a system of interrelated and uniquely identified computing devices that have the ability to transfer data through a network, without requiring human interactions. The IoT paradigm enables devices (mainly sensors and actuators) to communicate and perform jobs together, to share information and to coordinate decisions [44,45]. The IoT transforms these devices from being traditional to smart by exploiting their underlying technologies, such as ubiquitous and pervasive computing, embedded devices, communication technologies, sensor networks, and Internet protocols and applications [46]. Generally, in an IoT framework, three layers are considered:

1. *Perception Layer*: The lowest level of the three-layer architecture. This layer covers all physical devices, especially sensors and actuators, focused on measuring and collecting data and processing the information about the state of these devices. Furthermore, this layer is responsiblefor transmitting the information of the devices to the upper layers. For this reason, this layer is also frequently known as the *sensor layer*.
2. *Network Layer*: This layer is located in the middle of the IoT architecture and is also known as the *transmission layer*. The purpose of this layer is to receive the data from the bottom and the upper layer and transmit the information to the devices and applications divided across the layers finding the routers to perform the communication.
3. *Application Layer*: This is the upper layer of the IoT architecture and is the last layer. The aim of this layer is to receive the information provided by the network layer and to use this information in the services and applications developed to work with such data. Because this is the last layer where applications in servers are used, this layer is also known as the *business layer*.

A very important element in an IoT environment is the protocol to send the collected data from sensors over the three layers of the IoT architecture. One of the most used protocol is the Message Queue Telemetry Transport (MQTT) [47]. Internally, this protocol establishes connections based on TCP/IP where the connections remain open to be reused. The mechanics of this protocol are based on a publisher/subscriber architecture. The *publisher* is in charge of creating a topic and publishing the data associated with a *topic*. Both the data and the topic are managed on an intermediate element named the *broker*. The broker acts as a server that receives and sends data between the subscribers and the publishers. Finally, the *subscribers* receive data associated with a topic.

### 2.4. IEEE Std 1855-2016 and JFML

The IEEE Std 1855-2016 [48] was published in 2016 by the IEEE Computational Intelligence Society. This new standard introduces the Fuzzy Markup Language (FML) based on the syntax of the well-defined XML meta-language to represent FLS in a human-readable and hardware independent way [29]. It means that, thanks to FML, it is possible to implement the same FLS on different hardware architectures with a minimal effort and without additional design and implementation steps [49]. FML includes an extensible schema that defines the basic components of different types of FLS —including Mamdani, Tsukamoto and Takagi-Sugeno-Kang (TSK) [50]—but also the most recent AnYa [51]. The standard focuses on interoperability, i.e., the exchange of XML-based instances of FLS between various systems without taking care of hardware specifications, thus overcoming some limitations of embedded systems [32].

A FLS described in FML is characterized by five main components: (1) fuzzy knowledge base; (2) fuzzy rule base; (3) inference engine; (4) fuzzification subsystem; and (5) defuzzification subsystem. Furthermore, each component is described by a hierarchy of sub-components in order to fully specify a FLS. All such components are grouped into XML tags that also specify the IP address of devices that compute them, thus enabling networked interoperability among devices and sensors [52].

JFML is an open-source Java library that allows the design of FLS according to the IEEE Std 1855-2016 [31]. This library offers a complete implementation of all FLS types enclosed in the standard. Additionally, JFML includes a module to import/export FLS in accordance with different formats: FCL documents, the Predictive Model Markup Language (PMML) and the proprietary ‘*.fis’ format used by the Matlab Fuzzy Logic Toolbox.

JFML has been designed following the hierarchical structure of FML. It follows an object-oriented approach and a modular design based on the same labeled tree structure that FML uses to represent FLS. The library relies on Java Architecture for XML Binding (JAXB) to bind FML documents to Java representations, providing an efficient way for I/O operations. Accordingly, classes are organized in several interdependent packages, which implement specific features of FML following JAXB requirements. In addition, JFML includes the extension methods considered in the standard, thus facilitating the integration of changes in future releases of the standard. In addition, JFML can be accessed in Python 3.x through Py4JFML [53].

In addition, JFML can be used within an IoT infrastructure due to the IoT module which enables JFML to the Internet of Things capabilities. Hence, JFML is a powerful and scalable library that can be used together with an IoT infrastructure for designing FLS in the field of safety tasks.

## 3. Main Technologies Used in the Execution Phase

This section details some of the technologies that have been involved in IoT systems and identified in the literature as relevant for data acquisition during the execution phase in the life cycle of a construction project.

The most commonly used technologies, within the elements of an IoT system, we mainly consider those based on wireless communication as well as different sensors such as altimeters, anemometers, gyroscopes, accelerometers, temperature and humidity, etc. In addition, radio frequency identification (RFID) or global positioning systems (GPS or GNNS) are also considered. Sensors can measure the physical conditions of the world, such as position, occupancy, acceleration, velocity, movement, temperature, etc. As will be detailed below, they have been widely applied to monitor parameters including environmental status, occupant behavior, and energy usage inside buildings [54,55,56]. While Radio Frequency Identification (RFID) can, through electromagnetic fields, automatically identify and track tags attached to objects. Finally, the Global Positioning System (GPS) makes it possible to determine the position of people or objects with great precision.

Table 1 organizes several authors’ proposals as indicated above, firstly distinguishing the technologies (sensors, RFID and GPS) and secondly distinguishing whether the objective of the proposal is focused on health and safety aspects in the execution phase. As can be seen in the table, the most widely used technology is RFID, although the use of sensors has been winning greater acceptance in recent years. In relation to the phases of the work, most of the proposals focus on the execution phase and, specifically, to facilitate and improve safety management during the execution process. Within this task, which is very relevant in the construction sector due to the high accident rate, the proposals are mainly focused on identifying risks and alerting workers to avoid accidents [9,57,58]. Similarly, the control of some important activities such as planning [59] or the condition of concrete structures [60] have attracted the attention of the research community. In the maintenance phase, the proposals focus on energy efficiency management [61], planning in emergency situations [62] and occupancy estimation to adapt climatic conditions [63].

As can be seen in Table 1, the proposals dedicated to safety management mainly use RFID technology and sensors. All proposals present trends in the use of these technologies in the phases of construction projects that, in conjunction with the deployment under IOT infrastructure, show promising results.

## 4. IoT-JFML Proposal for the Risk of Falling Object Accidents

In order to improve the management of data collected by the sensors in the task of prevention the risk of falling object accidents, an IOT-JFML-based system was developed. To do this, on the one hand, we analyze several sensors/actuators from the perspective of their main characteristics that can be used from safety tasks. On the other hand, we propose an infrastructure for the system based on an integration of the JFML library, an IoT infrastructure based on MQTT and the proposed sensors/actuators.

### 4.1. Sensors Description

The main objective of the designed system is to prevent risk of falling object accidents on construction sites by considering data collected by several sensors in workplace areas during the execution phase. Information provided by the sensors is related to relevant variables in the risk of falling object accidents. The objects that commonly fall range from large items such as roof trusses and steel beams to small items such as fasteners, small hand tools and small particles from the loading crane.

The most relevant factors in the risk of falling objects are identified and monitored in the current approach. Wireless capabilities are required in this context because wired connections are not suitable for it.

- Use or non-use of a helmet. The adequate use of a helmet is a key factor in the risk of falling objects widely identified in the literature [77].
- Worker’s location. Identifying the exact location of the workers is one of the most important functions for accident prevention [78].
- Load crane location. Each crane has a load chart that, in short, specifies the crane’s capabilities. The location of the load in real-time is another factor to be considered for accident prevention [79].
- Worker’s altitude. Height is another important factor in the risk of falling object accidents. Many consequences of an accident depends on the altitude of the worker and fall and some authors identified the altitude as a good predictor of the overall outcome and chance of survival [80].
- Load crane altitude. In the same way, the altitude of the load is another key aspect of the severity of the accident [81].
- Safety barriers. Many accidents are explained by the lack of physical barrier elements. There is significant potential for accident prevention in the construction industry by systematic barrier management [82].
- Distance between workers and load crane. It is obvious that a worker placed near to the load crane is more likely to fall than another placed in away from that workplace. The distance from the load to the worker is another relevant variable monitored by the system [83].
- Wind velocity. Weather factors cannot be controlled in construction projects due to the outdoor nature of them [84]. Wind velocity has an adverse effect on construction sites [85]. Wind velocity was considered as a relevant variable too.

In order to measure, evaluate and control the described variables, the following sensors and devices, which are summarized in Table 2, have been included within the proposed real-time monitoring system.

All sensors are connected on ESP32 devices, which are designed for IoT applications. The ESP32 features a Hybrid Wi-Fi and Bluetooth Chip with in-built antenna switches, RF components, and power management modules.

For the helmet, an optical pulse sensor is embedded in the helmet for wearing detection. The optical pulse sensor measures pulse waves which are changes in the volume of a blood vessel that occur when the heart pumps blood. A set of *m* safety barriers ($Barrier_{1}$, $Barrier_{2}$,.... $Barrier_{m}$), each one composed of a set of gyroscopes, ($G_{i}^{1}$, $G_{i}^{2}$,... $G_{i}^{n}$) is also considered. In this case, the set of gyroscopes is distributed along the barrier and the orientation of axes and polarity of rotation can be used to determine if the barrier is fixed or not. On the other hand, an altimeter and a GNSS-antenna for GPS GLONASS 28dB are used to calculate the distance between workers and the load. An altimeter is also used for detecting when the worker or the load are placed in an elevated working place. In addition, an anemometer provides wind velocity at the workplace. A high velocity of wind could decrease the stability of the worker or load and then the risk of falling objects will be higher. Finally, a vibration sensor within a color LED is embedded into a wristband to advise the worker of the risk level of accident based on a color scale and the intensity of vibration. For instance, if the risk level is high, the color LED will be red and the vibration will be hard, if the risk level is medium, the color LED will be orange and the vibration will be medium or if the risk level is low, the color LED will be green and the vibration intensity will be soft.

On the basis of these sensors and their values, different working situations could be considered and the different risk levels can be estimated. For example, the BLE beacon attached to the headset, in combination with a BLE receiver detects if a worker is wearing a helmet or not. The receiver calculates the relative distance from the helmet to the worker based on a RSSI signal [75]. For example, if the distance between the BLE beacon and the receiver is lower than a value, the headset is considered as being attached to the worker. Taking these situations into consideration, sensors are distributed through the workplace by considering expert knowledge from the safety in construction sector. This distribution provides valuable information about working conditions in real time that will be used as input data in the IOT-JFML architecture defined in the following subsection.

### 4.2. Proposed Architecture

**Figure 1:**
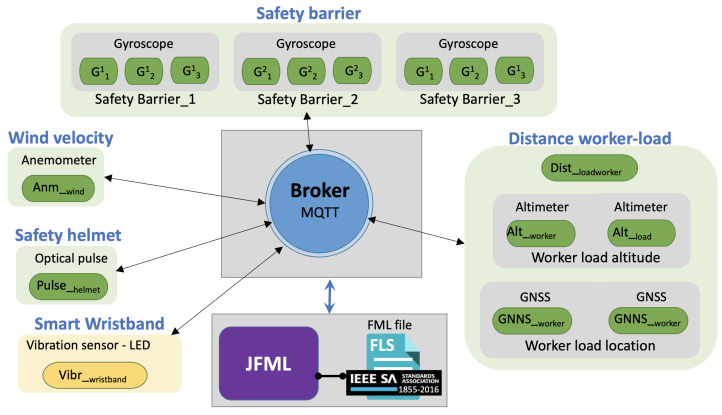
IOT-JFML architecture.

The JFML library and the new IoT module offer a complete implementation of an IoT infrastructure to carry out intelligent IoT solutions under the IEEE std 1855-2016. These capabilities are extended to the design and implementation of the previous falling object accidents proposal. Figure 1 shows an overview of the proposed architecture.

The main elements included in this implementation are the sensors/actuators, the broker, the JFML instance and the FML file that represents expert knowledge according to the IEEE std 1855-2016 for the fall object accidents problem. In general, sensors/actuators provide data that pass through the broker which are used for the JFML to make the inference according to the expert knowledge represented in the FML file.

The broker, which implements the MQTT protocol, is a Mosquitto instance [47] while the JFML library can be run on a remote computer or on the cloud. This architecture allows communication between all the elements due to the broker and the wireless capabilities. In the following section, we summarize the behaviour of this communication procedure:

1. Sensors provide data, which are published into “input” topics. In consequence, they must be associated with input variables. For example, the altimeter sensor for the worker ($Alt_{worker}$) publishes data into the topic “*input/Alt_worker*”, the anemometer sensor $Anm_{wind}$ publishes data into the topic “‘*input/Anm_wind*”, etc.
2. JFML is subscribed to all input topics to receive input data from the sensors and to assign them to the input variables. These input variables are defined in the FLS (represented in the FML file according to the IEEE std 1855-2016). For examxple, JFML is subscribed to the topics “‘*input/Alt_worker*”, “‘*input/Anm*’, etc. to receive data from the sensors $Alt_{worker}$ and $Anm_{wind}$, respectively. These sensors are associated with the input variables *worker altitude* and *wind velocity*, respectively. Hence, all input variables receive data from sensors.
3. When all of the sensors have published their data and JFML has assigned these values to the input variables, the inference is carried out. Rules are fired according to the input values and the rule base defined in the FML file.
4. Once the inference process is finished, the output variables obtain values from the corresponding defuzzification method. Then, JFML publishes these values to “output” topics. For example, the value of the output variable *smart wristband* is published by JFML into the topic “*output/wristband*”.
5. Actuators receive data so they are subscribed to “output” topics. In consequence, they must be associated with output variables. For example, the LED indicator and the vibration sensor Vibr are subscribed to the topic “*output/wristband*” to receive data from the output variable *smart wristband*.

## 5. Case Study

To illustrate the potential of this proposal in the prevention of falling object accidents on construction sites, a case study was developed. Specifically, a case focused on a building project and the execution phase where expert knowledge in the falling object accidents prevention are considered. In Section 5.1, the FLS by considering the expert knowledge from an expert panel is defined while in Section 5.2 the proposed FLS is modeled according to the IEEE 1855-2016. Finally, some illustrative results as two possible scenarios are presented in Section 5.3.

### 5.1. Defining the Fuzzy Logic System

**Figure 2:**
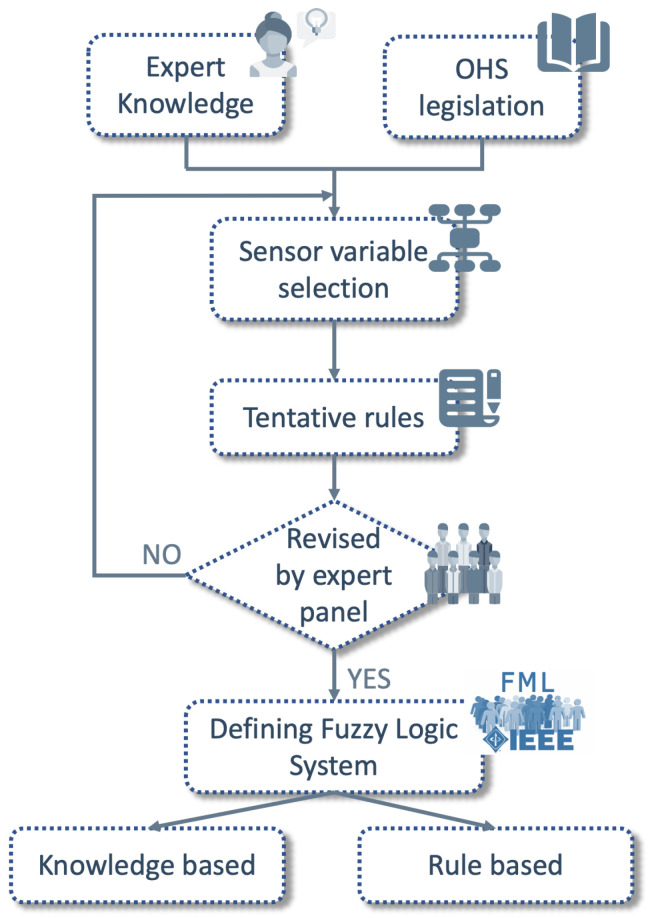
Methodology to represent the expert knowledge from a panel of experts.

Several working situations can be considered by relating them to risk levels using the proposed IOT-JFML architecture. Notice that these situations are involved to gradual and imprecise concepts both the variables and the situations. For example, the wind velocity can be low, medium or high depending on the gradual values provided by the anemometer. Or in the situation on the risk level, if the distance between the load crane and the worker is approximately lower than 1 m, the risk of falling object accident is very high. Hence, to represent this expert knowledge a Fuzzy Logic System is defined. In order to model the expert knowledge, a methodology has been followed in which a panel of experts has been involved. Figure 2 illustrates the flowchart of the proposed methodology.

Firstly, expert knowledge is brought together with occupational health and safety legislation in order to select variables to support the problem at hand. Based on the information gathered, tentative rules are proposed and reviewed by a panel of experts in this field of knowledge. If the experts do not validate the rules, the process is initiated in order to propose new sensors. However, if the proposal is validated, a fuzzy logic system is defined. For this purpose, firstly, the knowledge base is defined with several fuzzy variables and their fuzzy terms (Section 5.1.1). Secondly, the rule base is also defined considering the expert knowledge and the relationships between the fuzzy variables (Section 5.1.2).

#### 5.1.1. Defining the Knowledge Base

Several fuzzy variables which are related by some sensors/actuators are defined. Four input and one output variables were considered:

- *Safety helmet* represents the use of the helmet by the worker. It is an input variable defined by the singletons terms: *“Wearing”*, *“Not wearing”*.
- *Distance worker-load* represents the distance between the worker and the load crane. This variable is an aggregated variable that is calculated by means of both the altitude and the position of the load and the worker. It is an input variable composed of the fuzzy terms *“Near”*, *“Medium”* and *“Far”* in the domain [0,50] and represented in meters.
- *Safety barrier i* represents the correct placement of the barrier *i*. We consider 3 barriers in this research. Each variable is an input variable defined by the singletons terms: *“Installed”*, *“Uninstalled”*.
- *Wind velocity* represents the speed of the wind in the workerplace. It is an input variable defined by the fuzzy terms *“Low”*, *“Medium”* or *“High”* in the domain [0,120] and represented in km/h.
- *Smart wristband* represents the level of falling object accident risk. It is an output variable defined by the fuzzy terms *“Low Risk”*, *“Medium Risk”*, *“High Risk”* and *“Very High Risk”* in the domain [0,10].

#### 5.1.2. Defining the Rule Base

**Figure 3:**
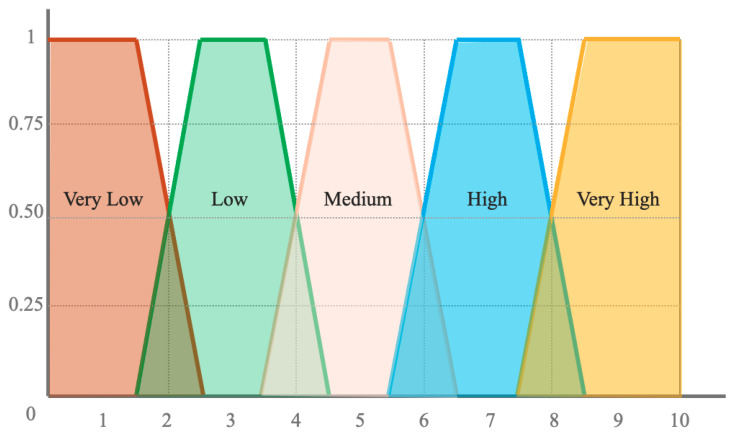
Definition of fuzzy risk levels.

Expert knowledge of the falling object accidents risk in a construction scenario in the form of rules is considered in order to determine the level of risk. A panel of experts defined different risk levels according to their experience of worker safety. They use five levels of risk labeled by imprecise terms such as very low, low, medium, high and very high risk. Based on this, five fuzzy sets were defined in the domain [0,10] in the form represented in Figure 3.

In addition, the expert panel proposed several rules to model risk situations in the execution phase. Specifically, they proposed 14 rules that are represented as pseudo-code in the following.

1.     IF
          Safety barrier IS Installed AND
          Safety helmet IS Wearing AND
          Wind velocity IS Low
      THEN
          Smart Wristband IS Low Risk
2.     IF
          Safety barrier IS Installed AND
          Safety helmet IS Not wearing
      THEN
          Smart Wristband IS Medium Risk
3.     IF
          Distance worker-load IS Near AND
          Safety helmet IS Not wearing
      THEN
          Smart Wristband IS Very High Risk
4.     IF
          Distance worker-load IS Medium AND
          Safety helmet IS Not wearing
      THEN
          Smart Wristband IS High Risk
5.     IF
          Distance worker-load IS Medium AND
          Safety helmet IS Wearing
      THEN
          Smart Wristband IS Medium Risk
6.     IF
          Safety barrier IS Uninstalled AND
          Distance worker-load IS Far AND
          Safety helmet IS Wearing
      THEN
          Smart Wristband IS Low Risk
7.     IF
          Safety barrier IS Installed AND
          Distance worker-load IS Far AND
          Safety helmet IS Wearing
      THEN
          Smart Wristband IS Very Low Risk
8.     IF
          Distance worker-load IS Far AND
          Wind velocity IS High
      THEN
          Smart Wristband IS High Risk
9.     IF
          Distance worker-load IS Near AND
          Wind velocity IS High
      THEN
          Smart Wristband IS Very High Risk
10.     IF
          Distance worker-load IS Medium AND
          Wind velocity IS High
      THEN
          Smart Wristband IS Very High Risk
11.     IF
          Distance worker-load IS Near AND
          Wind velocity IS Medium
      THEN
          Smart Wristband IS High Risk
12.     IF
          Distance worker-load IS Near AND
          Wind velocity IS Medium AND
          Safety helmet IS Wearing
      THEN
          Smart Wristband IS Medium Risk
13.     IF
          Distance worker-load IS Medium AND
          Wind velocity IS Medium AND
          Safety helmet IS Wearing
      THEN
          Smart Wristband IS Low Risk
14.     IF
          Distance worker-load IS Medium AND
          Wind velocity IS Medium AND
          Safety helmet IS Wearing AND
          Safety barrier IS Installed
      THEN
          Smart Wristband IS Very Low Risk

### 5.2. Fuzzy Logic System According to the IEEE 1855-2016

All of the previous rules and fuzzy variables have been represented in a FML file according to the IEEE std 1855-2016 specifications. As illustrative example, some parts of the file are shown in the Listing 1 although the complete FML file can be downloaded from the official JFML website http://www.uco.es/JFML (accessed on 15 July 2021). Knowledge base description provided by JFML is shown in the Listing 2.

### 5.3. Some Illustrative Results

In this subsection, some examples are detailed as simulations of real situations in a construction workplace during the execution phase by using the IOT-JFML proposal for falling object risk. As an illustrative example, we consider a scenario for material loading tasks.

In addition, an expert panel, comprising 10 persons (five professionals from construction companies and five academic researchers), evaluated the results. In these examples, the used sensors provide data, JFML receive them and the actuators act according to the output values provided by both the JFML and the FLS for falling object risk (defined in the previous section).

The performance of tests in real scenarios was not possible because of ethical and safety reasons. Logically, it is not safe to get out the worker helmet, or to place the worker near to the load, in order to test the possible values of the different variables. Only virtual scenarios were proposed to evaluate the system assessed by the expert panel.

#### 5.3.1. Case 1: Low Risk Scenario

**Figure 4:**
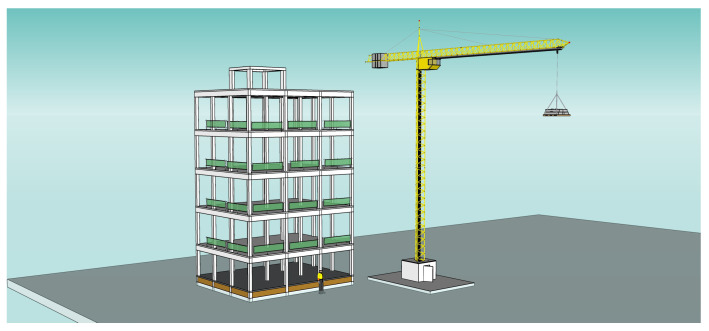
Visual example of material loading tasks: Low risk.

In many construction projects, material loading tasks are affected by the risk of falling to a lower level. For instance, the manual assembly of formwork structures, to place the slab formwork of the building for the next level. It should be noted that formwork activity on construction sites has been identified in the literature as an important source of occupational accidents, by different authors [91]. This case illustrated an example of this scenario where the worker is situated far from the load crane, the barriers are installed and there is no wind. Figure 4 shows a graphical representation of this scenario.

The altitude of the load crane was approximately 25 m, the worker is situated not so far from the load crane, all the safety barriers are installed and the wind velocity was estimated to be low with a value of 10 km/h. The worker is wearing the safety helmet.

In this situation, and taking into account these values as input data, the risk value provided by the IoT-JFML system was 3.9999967. In this case (see Listing 3), the smart wristband warns the worker with a soft vibration and illuminates the green LED indicating that the risk of an accident is low. This scenario fired Rule 1 and Rule 5 corresponding to the safety barriers are installed, the worker is wearing the helmet and the distance of the worker to the load is medium; as a consequence, the risk was low. This result is in accordance with the expert panel for this scenario; they agreed.

#### 5.3.2. Case 2: Medium Risk Scenario

**Figure 5:**
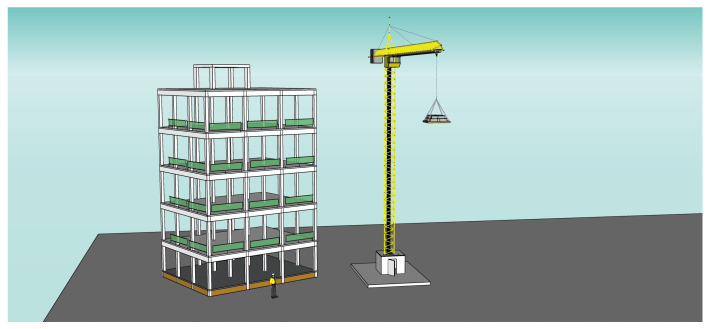
Visual example of material loading tasks: Medium risk.

In the second scenario analyzed, a similar scenario to the previous one is described but in this case, the load is nearer than the previous scenario and little wind appears. Figure 5 illustrates a visual example of this scenario.

In this situation, the risk considered by the expert panel is medium. To reproduce this scenario, the wind velocity was considered to be 40 km/h, the safety helmet is wearing and the distance from the worker to the load was 11 m. The risk value provided by the IoT-JFML system was increased with respect to the previous scenario, concretely the risk value was 6.72879. This scenario fires Rule 5 and Rule 9 with a weight of 10% corresponding to a medium distance and the wind velocity is high so the risk is medium (see Listing 4). Again, this result was accepted by the expert panel to be representative of this scenario.

#### 5.3.3. Case 3: Very High Risk Scenario

**Figure 6:**
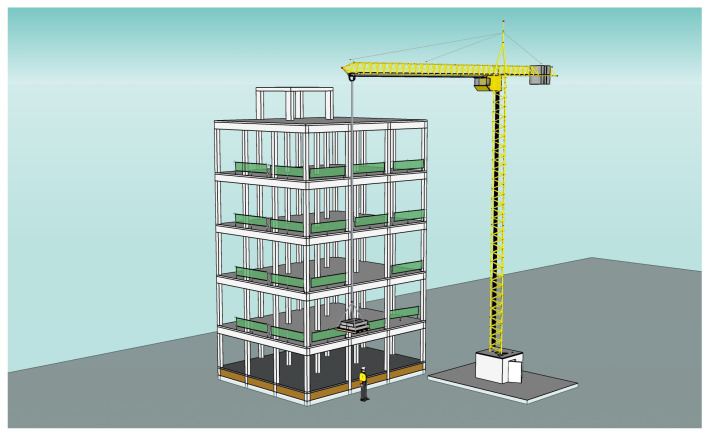
Visual example of material loading tasks: Very High risk.

In the third scenario, the presence of scaffolds and the worker near the load crane on a windy day were studied. Scaffolds can be defined as a temporary elevated platform, either supported or mounted. They are very useful when carrying out works on facades and upper floor construction or maintenance. Many scaffolding accidents are likely to be fatal due to the specific characteristics of the works [92]. In consequence, other authors estimated the occupational risk associated with working on scaffoldings, based on the analysis of recorded accidents, identification of the most common scenarios, and the estimation of the probability of their occurrence [93]. Then, one of the proposed scenarios was associated with scaffolding and materials movements (Figure 6).

In the current example, the worker was placed near to the load crane on a windy day. The wind velocity was considered to be 50 km/h. The risk value provided by the IOT-JFML system was 8.979336 and Rule 3 and Rule 10 were fired. In this case (see Listing 5), the level of risk is very high due to the near distance from the worker to the load and the wind velocity. In the same way, the expert panel agreed with the results from this scenario.

## 6. Conclusions

The life cycle of construction projects is a long process involving different stages with different objectives. Although the construction industry has always had a very traditional nature involving many small companies and where technology has not been very present, today new approaches such as the Internet of Things are playing a key role in improving the decision-making process.

This paper proposed an IoT infrastructure combined with the Fuzzy Markup Language on JFML for on-site construction safety—the IOT-JFML system for falling objects. This system enables detection and management of this mentioned risk during the construction process by means of several sensors, an actuator, a broker based on the MQTT protocol, an instance of JFML and an FML file.

In particular, we focus on the execution phase, as this is the phase where there are most risks and where most accidents occur. One of the most frequent risks due to the characteristics of the construction process is falling objects. A complete analysis has been carried out with the aim of identifying the relevant factors for this risk. Then, different sensors have been proposed to measure these factors (beacon, gyroscope, altimeter, anemometer, etc.). To model the risk situation and to evaluate the proposal a panel of experts comprising 10 persons (f9ve professionals from construction companies and five academic researchers) were consulted. Following a methodology for determining a Fuzzy Logic Systems where the panel of experts has been involved, four input and one output fuzzy variables and 15 fuzzy rules which have been considered to design a Fuzzy Logic System (FLS) for this risk problem.

The main elements included in this system implementation are the sensors/actuators, the broker, the JFML instance and the FML file that represents expert knowledge according to the IEEE std 1855-2016 for the fall object accidents problem. Sensors provide data that pass through the broker which are used for the JFML to make the inference according to the expert knowledge represented in the FML file. Then, a vibration sensor within a color LED are embedded into a wristband to advise the worker of the risk level of accident on the basis of a color scale and the intensity of vibration.

Finally, in order to illustrate the potential of this proposal, three habitual working scenarios are detailed as simulations of real situations in a construction workplace during the execution phase by using the IOT-JFML proposal for falling object risk. As an illustrative example, we consider a scenario for material loading tasks. The proposed architecture is flexible and scalable so, in future research, it could be extended to multiple workers in different situations. In the case of multiple tasks with multiple workers, the information obtained will help to coordinate construction safety practices.

The main advantage of the proposed system is the ability to assess risk levels for construction workers in real-time. In traditional risk assessment, risks are identified prior to the start of construction works. However, due to the dynamic nature of the construction industry, the risk of falling objects can constantly change. In this sense, our proposal allows for continuous monitoring of workers, offering the opportunity to update risk levels in real-time. Our proposal could be used as input data for the establishment of active BIM environments that are currently under development, but are expected to be used effectively as a decision-making tool for construction project management, by linking the two approaches to reduce the risk of falling objects during the construction process.

Finally, the proposed architecture could have an important implication also in other real world applications, not only for falling object risk in construction phases. For example, the proposed architecture based on an IoT infrastructure combined with the Fuzzy Markup Language on JFML could be equipped with a different set of sensors to address forensic science scenarios such as the analysis of crime scenes and the automatic reconstruction of crime dynamics [94,95].

## Figures and Tables

**Table 1:** Proposals according to technologies and project life cycle phases.

	Technologies					
	Accelerometer	Beacons	Gyroscope	Optical Pulse	RFID	GPS
Wu et al. [41]					x	
Teizer and Cheng [64]						x
Park et al. [65]					x	
Nath et al. [66]	x		x		x	
Fang et al. [67]					x	x
Wang and Razavi [68]						x
Barro-Torres et al. [69]					x	
Park et al. [70]		x				
Lee et al. [71]	x					
Valero et al. [72]			x			
Park et al. [73]	x	x	x			
Kanan et al. [9]					x	
Antwi-Afari and Li [74]	x					
de Gabriel et al. [75]		x			x	
Chae and Yoshida [57]					x	
Yang et al. [43]				x	x	
Robinson et al. [76]	x			x	x	

**Table 2:** Variables and sensors for the system.

Variable Name	Sensor Type	Sensor Notation
Safety Helmet	Optical Pulse [86]	$Pulse_{helmet}$
Worker Location	GNSS [87]	$GNNS_{worker}$
Load Location	GNSS [87]	$GNNS_{load}$
Worker Altitude	Altimeter [88]	$Alt_{worker}$
Load Altitude	Altimeter [88]	$Alt_{load}$
Safety barrier	Gyroscope [89]	$Barrier_{i}$
Distance worker-load	Altimeter [88]/GNSS [87]	$Dist_{loadworker}$
Wind velocity	Anemometer [90]	$Anm_{wind}$
Smart wristband	Vibration [86]/Color LED	Vibr

**Listing 1:** Some part of the FML file according to the IEEE std 1855-2016 designed for the Falling Object Risk.

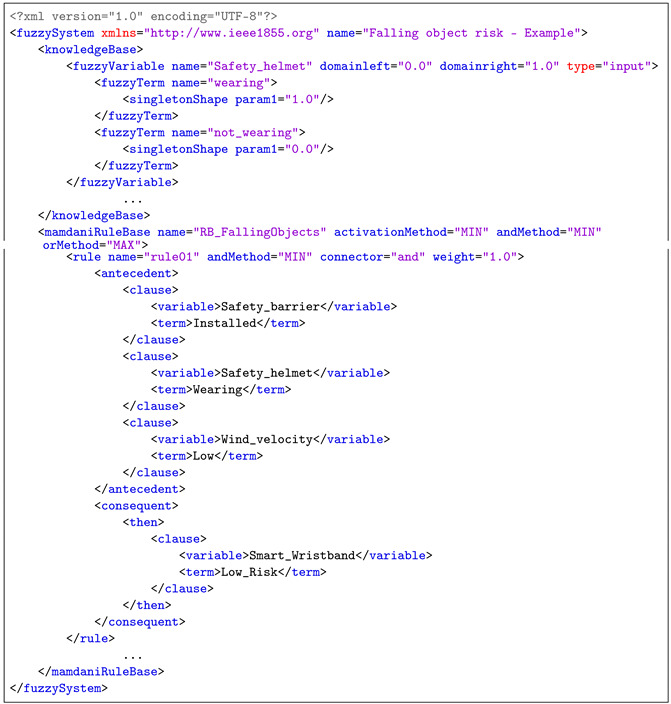

**Listing 2:** Knowledge base description provided by JFML.

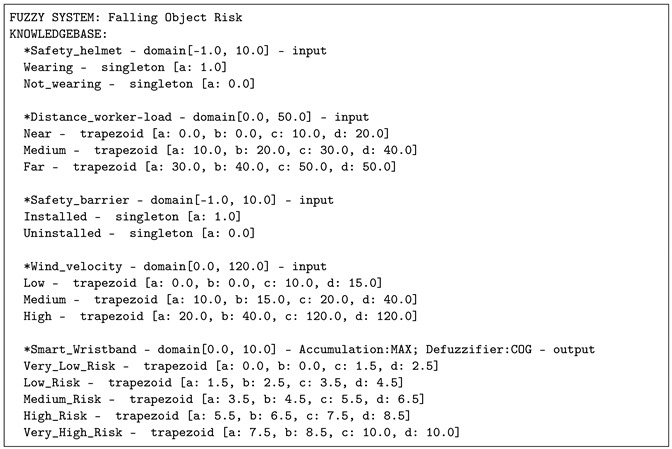

**Listing 3:** Case 1: Results provided by the IOT-JFML system for the scenario 1 (low risk).

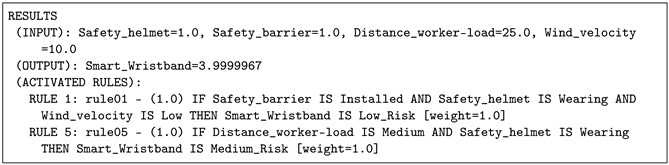

**Listing 4:** Case 2: Results provided by the IOT-JFML system for the scenario 2 (medium risk).

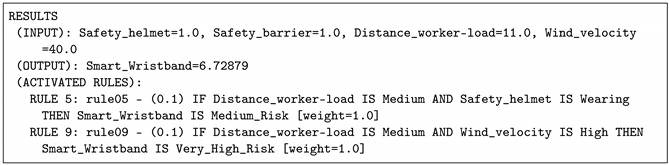

**Listing 5:** Case 3: Results provided by the IOT-JFML system for the scenario 3 (very high risk).

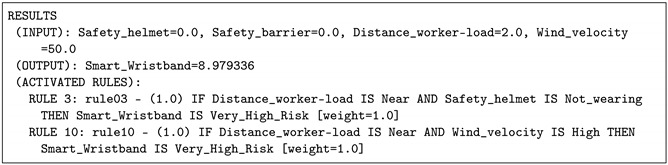

## Data Availability

Not applicable.

## Abbreviations

BIM	Building Information Modeling
BLE	Bluetooth Low Energy
FLSs	Fuzzy Logic Systems
FML	Fuzzy Markup Language
GPS	Global Positioning System
ICT	Information and Communication Technologies
IEEE-CIS	IEEE Computational Intelligence Society
IoT	Internet of Things
JAXB	Java Architecture for XML Binding
JFML	Java Fuzzy Markup Language
MQTT	Message Query Telemetry Transport
PMML	Predictive Model Markup Language
PPE	Personal Protective Equipment
RFID	Radio Frequency Identification
TCP/IP	Transmission Control Protocol/Internet Protocol
WSDs	Wearable sensing devices
XML	eXtensible Markup Language
